# Fatal Status Epilepticus in Dravet Syndrome

**DOI:** 10.3390/brainsci10110889

**Published:** 2020-11-23

**Authors:** Paola De Liso, Virginia Pironi, Massimo Mastrangelo, Domenica Battaglia, Dana Craiu, Marina Trivisano, Nicola Specchio, Rima Nabbout, Federico Vigevano

**Affiliations:** 1 Department of Neuroscience, Bambino Gesù Children’s Hospital, IRCCS, Full Member of European Reference Network EpiCARE, 00165 Rome, Italy; paola.deliso@opbg.net (P.D.L.); virginia.pironi@gmail.com (V.P.); marina.trivisano@opbg.net (M.T.); nicola.specchio@opbg.net (N.S.); 2 Center for Rare Diseases and Birth Defects, Department of Woman and Child Health, Institute of Pediatrics, Policlinico Universitario Gemelli Foundation, Catholic University of Rome, 00168 Rome, Italy; 3 Paediatric Neurology Unit, V. Buzzi Hospital, A.O. ICP, 20019 Milan, Italy; massimo.mastrangelo@asst-fbf-sacco.it; 4 Department of Child Neurology and Psychiatry, Policlinico Universitario Gemelli Foundation, Catholic University of Rome, 00153 Rome, Italy; domenicaimmacolata.battaglia@policlinicogemelli.it; 5 Department of Neurology, Paediatric Neurology, Psychiatry, Neurosurgery, “Carol Davila” University of Medicine of Bucharest, Full Member of European Reference Network EpiCARE, 050474 Bucharest, Romania; dcraiu@yahoo.com; 6 Centre for Rare Epilepsies, Department of Paediatric Neurology, Necker-Enfants Malades Hospital, Imagine Institute, INSERMU1163, Paris Descartes University, Full Member of European Reference Network EpiCARE, 75006 Paris, France; rimanabbout@yahoo.com

**Keywords:** Dravet syndrome, status epilepticus, acute encephalopathy, SCN1A, cause of death

## Abstract

Dravet Syndrome (DS) is burdened by high epilepsy-related premature mortality due to status epilepticus (SE). We surveyed centres within Europe through the Dravet Italia Onlus and EpiCARE network (European Reference Network for Rare and Complex Epilepsies). We collated responses on seven DS SCN1A+ patients who died following refractory SE (mean age 6.9 year, range 1.3–23.4 year); six were on valproate, clobazam, and stiripentol. All patients had previous SE. Fatal SE was always triggered by fever: either respiratory infection or one case of hexavalent vaccination. SE lasted between 80 min and 9 h and all patients received IV benzodiazepines. Four patients died during or within hours of SE; in three patients, SE was followed by coma with death occurring after 13–60 days. Our survey supports the hypothesis that unresponsive fever is a core characteristic feature of acute encephalopathy. We highlight the need for management protocols for prolonged seizures and SE in DS.

## 1. Introduction

Dravet syndrome (DS) is a severe developmental and epileptic encephalopathy (DEE) that is usually due to *SCN1A* genetic variants for the voltage-gated sodium channel Nav1.1. DS is characterized by onset of hemiclonic or generalised fever-triggered seizures within the first year of birth followed by afebrile multiple seizure types. The majority of patients have repeated episodes of status epilepticus (SE), often triggered by fever [1].

Mortality is high with DS (range 5.75–10%) [1,2,3], with sudden unexpected death in epilepsy (SUDEP) seemingly more frequent than intractable SE (49% versus 32%) [3]. However, death due to intractable SE is frequent among patients younger than 10 years old (86%), with a peak around 6 years old [2]. SE-related mortality risk might be due to an acute encephalopathy (AE), a potentially fatal complication in children with DS. SE has been reported as the initial manifestation of AE in children with DS, followed by prolonged consciousness disturbance, severe neurological sequalae, or death [4].

Deaths and long-term sequelae due to SE and AE have been increasingly reported over the past decade [4,5,6,7]. Incidence of AE was estimated as between 5.6% and 7.6% from several DS case series [4,7]. Fatal SE and AE usually occur in patients who have already experienced SE without sequelae [4,6,7]. Brain magnetic resonance imaging (MRI) studies performed in closed temporal proximity to the SE are rare but revealed diffuse cerebral oedema [4,6,7].

Possible predictive factors for SE with AE include early age at epilepsy onset (<6 m), epilepsy severity and recurrent SE, age under five years [7,8], and truncating *SCN1A* mutation [6]. Several hypotheses explain mechanisms leading to death: hemodynamic failure due to duration of SE, cardiac rhythm abnormalities due to *SCN1A* channelopathy, and effect of drugs for SE on cerebral blood flow [5]. Most authors agreed that highly intractable fever was a constant feature in AE [4,6,7]. Chronic use of benzodiazepines has been considered responsible for SE refractoriness; however, its role was not confirmed [7].

Following the death of two of our patients during a febrile refractory SE, we designed a survey with the aim of identifying causative and predisposing factors to fatal SE in DS.

## 2. Materials and Methods

We launched an international request through the Dravet Italia Onlus patients association and the 28 centres affiliated to the European Reference Network on Rare and Complex Epilepsies (EpiCARE) to identify patients with DS who died following generalized convulsive status epilepticus (GCSE). In June 2018, an electronic form was sent to all centres that reported at least one case to collect clinical features, history of epilepsy, and data about the SE leading to death. Patients who died from SUDEP or causes other than SE, or had insufficient clinical data were excluded from our analysis. Patients’ data were collected anonymously (Table 1). The study was approved by the local Ethics Committees.

## 3. Results

Four Dravet Italia Onlus centres and six EpiCARE centres responded to our request. Survey forms were returned for ten patients. Seven patients (4 females) fulfilled the inclusion criteria. Four patients came from Italy (3 centres), two from France (1 centre), and one from Romania.

The median age at death was 2.5 years (range 1.3–23.4 year, mean 6.9, SD 8.1 year). All patients had a *SCN1A* genetic variant: three truncating, three missense, and one frameshift (Table 1). Mean age at epilepsy-onset (*n* = 4) was five months (range 4–7 m). During the six months before the fatal SE, two patients were seizure-free, two had rare tonic-clonic seizures, and three had monthly seizures. All patients received polytherapy: six had valproate (VPA) combined with clobazam (CLB) and stiripentol (STP); one had VPA and Topiramate (TPM). All patients had a seizure action-plan for out-of-hospital management; caregivers were instructed on early initiation of paracetamol in case of fever and home rescue-medication with benzodiazepines in case of prolonged or clustering seizures. All patients previously experienced SE, with four having experienced multiple SE events. Previous SE were treated successfully with benzodiazepines for all patients, with three having also received phenytoin or propofol.

In all cases, SE was triggered by fever and body temperature increased progressively during the SE. Mean (SD) temperature was 40 (1.8) °C (range 38–42 °C). In six patients, fever was associated with respiratory symptoms. In one patient, temperature increased 10 h after vaccination (single dose of DTPa-HBV-IPV/Hib vaccine). Duration of the GCSE ranged from 80 min to 9 h (*n* = 4). Four patients died within hours of convulsive SE (Table 1). With three patients (#3, #4, #6), SE was followed by a deep coma-state and death after several days (13, 60, and 25 d, respectively). All patients died in hospital.

Following the onset of seizures that led to SE, all patients but one received benzodiazepines within 3 to 5 min, and all patients received intravenous anti-seizure medications within 30 min. Four patients received rectal or oromucosal benzodiazepines in an out-of-hospital setting, and all patients had IV benzodiazepines in hospital (Table 1). Phenytoin and propofol were each given to three patients. Patient #6 died 25 days after receiving ketogenic diet as adjunctive treatment. Compared with previous episodes for each patient, no remarkable differences were evident in triggers of SE, type of treatment, or time interval to treatment.

**Figure 1:**
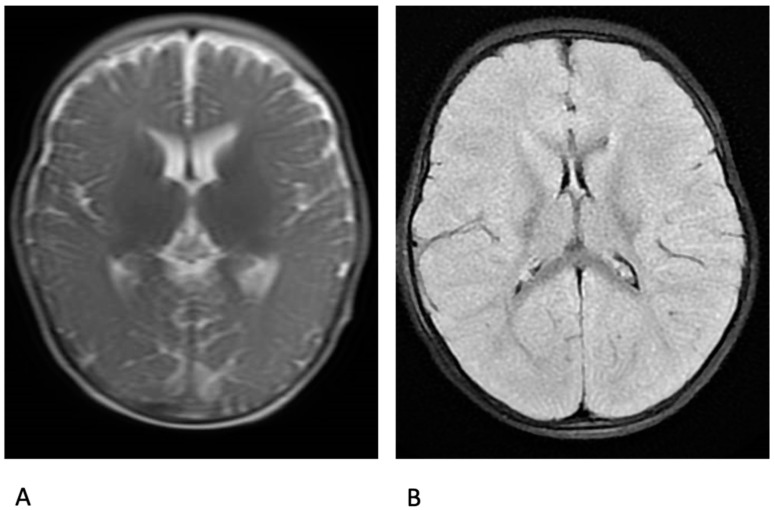
Brain MR of patient #6. (**A**). Axial T2 weighted MR at 6 months of age with no abnormalities. (**B**). Axial T1 weighted MR after 20 h from SE onset, showing diffuse brain edema.

Three patients (#4, #6, #7) underwent a neuroimaging study soon after SE onset (2 had brain MRI and #7 had computerised tomography), which each showed diffuse cerebral oedema (Figure 1). Patient #6 also had brain MRI 20 days after SE onset, which showed cortical and subcortical atrophy. Although requested on the survey form, no data from autopsy were available.

## 4. Discussion

We describe seven patients with DS who died following SE. Death followed two distinct developments: four patients died during or within a few hours of SE beginning; the SE of three patients evolved into a coma-like state until death several days later. The latter progression to death has been described more frequently in the literature (Table 2).

Cerebral oedema, persistent seizure activity, and hypoxia might explain the ultimate cause of death in patients with DS and fatal SE. According to reported data, a massive brain oedema is evident during the early phase of AE, quickly leading to death or severe brain damage. Neuroradiologic findings from the three of our patients who received a brain MRI in the early phase did indeed have evidence of brain oedema.

Several hypotheses for development of cerebral oedema in patients with DS have been proposed, though a complex of these mechanisms could underlie the pathogenesis. Ultimately, cerebral oedema in patients with epilepsy might be due to the excessive influx of sodium and calcium ions into neurons [9]. That hyperthermia induces epileptic seizures in DS is well known, and these might lead to post-ictal respiratory failure and cardiac arrest, and subsequently to neuronal sodium-ion influx. The latter could also be directly due to the mutations in *SCN1A*.

SE is known to be the second most common cause of death in DS, though deaths from SE occur more frequently in patients younger than 10 years old. Several patients have been reported to have died from SE in previous case series [2,4,6,7,10,11,12,13,14,15,16], but few cases had their clinical features described (Table 2) [4,6,7].

Demographic data of our patients are consistent with those previously reported and support that children under the age of six years have a higher risk of SE with AE [7,8]. However, one of our patients (#1) was a young adult, which we believe is only the second patient reported in literature to have died from SE in adulthood, the other being reported by Genton et al. in less detail [11]. Similarly, a 17 year-old from the UK was reported to have died from SE, but clinical data are unavailable for comparison [12].

All our patients were treated with anti-seizure medications (ASMs) recommended for DS. None of the patients was on known potentially aggravating ASMs. Propofol was used for the treatment of SE in only three out of seven patients, so—if a putative contributor to cerebral oedema—this was not a risk factor common to all patients. Moreover, some data from animals exposed to propofol have been reported to support attenuation of cerebral oedema after transient cerebral ischaemia [17].

At the time of the SE leading to death, patients had variable control of seizures; moreover, two patients were seizure-free in the preceding 6 months. These data support that death can occur unpredictably, including in patients with seemingly good seizure-control. Six of our patients were taking an ASM regimen that included STP; in contrast, only one patient was on STP in previously reported series [6]. Even if STP was demonstrated to decrease significantly prolonged seizures and motor SE frequency [18], it seems that it does not prevent the occurrence of fatal SE. All patients had previously received appropriate treatment based on the current recommendations; however, we acknowledge that none was treated with newly approved drugs for DS, such as cannabidiol and fenfluramine. Based on our observation, we suggest an early use of new compounds that might be effective in reducing the recurrence of SE and potentially protect patients with DS from fatal evolution of SE.

High body temperature was reported as a key factor in the development of AE [4,6]. Presumed viral infections are frequently reported and these as a possible triggering factor is consistent with the AE pathogenesis reported by Mizuguchi and colleagues [19]. With Patient #5, fever was triggered by a second dose of hexavalent vaccine. Vaccination has been previously reported to be a trigger for febrile seizures with *SCN1A* genetic variants [20]. McIntosh et al. reported that 45% of DS patients who presented a first seizure in proximity of vaccination might experience a SE [21]. Tsuij et al. described a patient with DS with a SE associated with AE after influenza vaccination; fortunately, without a fatal outcome [22]. Recently, Deng et al. reported a fatal SE following vaccination in a 12 month-old boy with *SCN1A* genetic variant [23]; clinical phenotype of this patient was suggestive of ‘generalized epilepsy with febrile seizures plus’ (GEFS+) rather than DS. Notably, this patient had a febrile illness before vaccination, so other aetiological agents could plausibly have been involved in this catastrophic outcome. Therefore, we suggest particular attention is required during vaccination of patients with DS and, in our opinion, tailored recommendations are needed.

The role of fever and immune mechanisms should be further investigated to clarify if AE can be considered as a DS-specific complication or as the extreme spectrum of an immune-mediated disorder [19]. However, such a high mortality with SE or AE has not been described for other DEE that share with DS a sensitivity to fever, such as *PCDH19*-Girl Clustering Epilepsy [24]. Nevertheless, cases of death from SE and SUDEP are also reported with GEFS+, highlighting that death may also occur in patients with a less severe epileptic phenotype than DS [23,25].

The presence of sodium-channel variants may have further increased the SUDEP risk. Noteworthy is that *SCN1B* genetic variants have also been associated with cardiac arrhythmias, including Brugada and long QT-interval syndromes [26]. These data suggest that sodium-channel mutations might play a crucial role in causing AE and SUDEP [27].

With respect to emergency care, most patients died in local hospitals and time was minimal for transfer to the reference centre for rare epilepsies of their regional hospital; SE developed and progressed rapidly and did not allow time for referral. Hence, a SE-management protocol for DS should be available for patients and carers. Rapid access to specific recommendations for management of fever and prolonged or repetitive seizures in this syndrome should be available to emergency room physicians unfamiliar with this syndrome and some psychiatric intensive care-unit physicians away from the reference centres.

## 5. Conclusions

This retrospective analysis of patients who died during a SE supports—albeit weakly—the hypothesis that unresponsive fever is a core characteristic feature of AE. Treatment of fever should be part of the management plan for prolonged seizures in DS and families should be instructed on its management. Vaccination requires close monitoring [28].

Considering chronic treatment, current recommendations for the treatment of DS should be implemented with inclusion of newer compounds, since any protection of existing drugs against fatal events remains undiscovered. Since fatal SE is a rare, unpredictable event, increased effort to collect the few data available and request for post-mortem examination might enhance the understanding of the mechanisms of this acute event.

## Figures and Tables

**Table 1:** Clinical features, epilepsy history, and fatal status epilepticus.

Pt	Sex	Age at Epilepsy Onset (m)	Age at Death (y)	*SCN1A* Variant	Seizures during the 6 Months before Death	Ongoing ASMs	Previous SE	Treatment of Previous SE	Trigger of SE	SE Duration (min)	Body Temperature (°C)	Neuroimaging after SE Onset	SE Treatment
1	M	NA	23.4	c.5414-5415delTT (p.Phe1805Stop)	No seizures	VPA, CLB, STP, TPM, PER	Yes	DZP	Flu	120	42	np	Rectal DZP (at home), IV MDZ
2	M	4	2.5	c.17_18dupTT (p.Val7Leufsx86)	focal, 1/m, <10 min	VPA, TPM	Yes	DZP	NSFI	80	38	np	VPA, TPM
3	M	NA	4.8	c.664T>C (p.Arg222*)	GTC, 1-3/m, 8–30 min; focal, 2–3/m, 10–20 min	VPA, CLB, STP	Yes	DZP, MDZ	NSFI	NA	NA	np	PHT, DZP, MDZ, propofol
4	F	NA	11.9	c.2503T>A (p.Leu835Met)	GTC, rare, <10 min	VPA, CLB, STP	Yes	DZP, MDZ, PHT	Fever, respiratory distress, *ab ingestis* pneumoniae	NA	40	np	Rectal DZP (at home), IV MDZ, IV LEV, IV PHT
5	F	4	2.5	c.4934G>A (p.Arg1645Gln)	no seizures	VPA, CLB, STP	Yes	MDZ, Propofol	Fever, vaccination (DTPa-HBV-IPV-Hib)	150	38	np	Rectal DZP, IV MDZ, propofol
6	F	5	2.3	c.1837C>T (p.Arg613*)	GTC, monthly, long lasting; focal, rare, brief	VPA, CLB, STP, CLZ	Yes	MDZ, DZP, CLZ PHT	RSV infection	(25 days)	42	MRI: significant cytotoxic oedema in the grey matter and cortex	Oromucosal MDZ, CZP; PHT; MDZ; propofol; LEV; KD
7	F	7	1.3	c.4427A>G (p.Asn1476Ser)	GTC rare brief	VPA, CLB, STP, LEV	Yes	DZP, MDZ, CLZ	NSFI	540	40	CT scan: diffuse cerebral oedema	MDZ

ASMs, anti-seizure medications; CLB, clobazam; CLZ, clonazepam; CT, computerised tomography; DZP, diazepam; F, female; Flu, influenza-like symptoms; GTC, generalized tonic-clonic; KD, ketogenic diet; LEV, levetiracetam; M, male; MDZ, midazolam; MRI, magnetic resonance imaging; NA, not available; np, not performed; NSFI, nonspecific febrile illness; PER, perampanel; PHT, phenytoin; Pt, patient; RSV, respiratory syncytial virus; SE, status epilepticus; STP, stiripentol; TPM, topiramate; VPA, valproic acid.

**Table 2:** Published cases of death of patients with Dravet syndrome presenting acute encephalopathy during status epilepticus.

Author	Sex	Age at Epilepsy Onset (m)	Age at Death (y)	*SCN1A* Variant	History of SE	Seizures Frequency	Ongoing ASMs at Death	Fatal SE Trigger	Duration of SE (h)	Temp (°C)	Fatal SE Treatment
Okumura, 2012 [4]	M	3	4.4	R568X	3	monthly *	VPA, CZP, KBr	Flu	4	NA	DZP, PHT, TL MDZ
Okumura, 2012 [4]	F	3	1.1	R701X	0	none *	VPA, CZP, PB	NSFI	3	NA	DZP, MDZ, PHT, PTB, TP
Okumura, 2012 [4]	M	5	15.3	np	1	monthly *	VPA, ZNS, NZP	URI	1	NA	DZP, MDZ
Okumura, 2012 [4]	M	4	3.6	np	7	monthly *	VPA, ZNS, CLB	NSFI	5	NA	DZP, MDZ, PB, TP
Myers, 2017 [6]	NA	NA	5	c.5347G>A, p. Ala1783thr	several	seizure free **	VPA, TPM	URI, Flu (A+)	1.25	40	MDZ, PHT, TP
Myers, 2017 [6]	NA	NA	8	c.5741_5742delAA, p.Gln1914fs*1943	3	1–2/year	VPA, TPM	abdominal pain, diarrhoea	1.5	43.7	MDZ, PHT
Myers, 2017 [6]	NA	NA	11	c. 4633A>G	several	seizure free ^	VPA, TPM, STP	sore throat	2	41	MDZ, PB
Myers, 2017 [6]	NA	NA	5	c.4970G>A, p. Arg1657His	12	2 seizures ^^	LTG, VPA	viral URI	4	40	MDZ, DZP, PHT, TP, PB
Myers, 2017 [6]	NA	NA	0.8	c.3136delG, p.Asp1046Metfs*1055	0	1–2/m, long lasting	TPM, LEV	viral URI	1.5	40	CZP, MDZ, PHT
Tian, 2018 [7]	F	5	3	R101W	2–3/year	monthly	VPA, LEV	NA	2	39.3	NA
Tian, 2018 [7]	M	2.5	4	V422A	3–4/year	monthly	VPA, LEV	NA	2	40.5	NA
Tian, 2018 [7]	M	5.5	5	L556fsX	4–5/year	monthly	VPA, TPM	NA	4	40.4	NA
Tian, 2018 [7]	F	5	10	R612X	1–2/year	monthly	VPA, LEV, CZP	NA	2	39.5	NA
Tian, 2018 [7]	F	4	3	W1286X	4–5/year	monthly	VPA, LEV	NA	2	39.3	NA
Tian, 2018 [7]	F	5	5	A1783T	2–3/year	yearly	VPA, TPM	NA	3	40.6	NA
Tian, 2018 [7]	M	2	4.2	c659-1G>A	1–2/year	monthly	VPA, TPM, CZP	NA	3	39.5	NA
Tian, 2018 [7]	F	4	5.2	R1892X	2–3/year	monthly	VPA, TPM	NA	2	normal	NA
Tian, 2018 [7]	M	5	3.4	F1699S	2–3/year	monthly	VPA, LEV	NA	5	39.5	NA
Tian, 2018 [7]	M	5	1.3	Q1904X	2–3/year	yearly	VPA, TPM	NA	5	40.2	NA
Tian, 2018 [7]	F	3	2.7	S243Y	1–2/year	monthly	CZP, LEV, ZNS	NA	12	40.3	NA
Tian, 2018 [7]	F	4.5	3.3	I1810N	1–2/year	monthly	VPA, LEV	NA	2	39.5	NA

* Seizure frequency refers to the last 3 months before the acute encephalopathy (AE); ** Seizure frequency refers to the last 5 months before the AE; ^ Seizure frequency refers to the last 18 months before the AE; ^^ Seizure frequency refers to the last 9 months before the AE. AE, acute encephalopathy; ASMs, anti-seizure medications; CLB, clobazam; CZP, clonazepam; DZP, diazepam; F, female; Flu, influenza-like symptoms; KBr, potassium bromide; LEV, levetiracetam; LTG, lamotrigine; M, male; MDZ, midazolam; NA, not available; np, not performed; NSFI, nonspecific febrile illness; PB, phenobarbital; PER, perampanel; PHT, phenytoin; SE, status epilepticus; STP, stiripentol; TL, thiamylal; TP, thiopental; TPM, topiramate; URI, upper respiratory tract infection; VPA, valproic acid; ZNS, zonisamide.

## Abbreviations

AE	acute encephalopathy
ASMs	anti-seizure medications
DEE	developmental and epileptic encephalopathy
DS	Dravet syndrome
EpiCARE	European Reference Network on Rare and Complex Epilepsies
GEFS+	generalized epilepsy with febrile seizures plus
GCSE	generalized convulsive status epileptic
MRI	magnetic resonance imaging
SE	status epilepticus
SUDEP	sudden unexpected death in epilepsy
